# Prefrontal Circuitry Abnormalities and Cognitive Impairment in Adolescents with Early- Onset Psychosis

**DOI:** 10.21203/rs.3.rs-10021774/v1

**Published:** 2026-06-28

**Authors:** Hanne Van Der Heijden, Saad Rahmat, Amanda Cao, Raquel van Gool, Merve Koç Yekedüz, Lise Vrolix, Talia Barrett, Edin T. Randall, Itamar Ronen, James Barnacle, Maya Golden, Maria Goldman, Benjamin Lu, Joyce C. Chang, Jason M. Fogler, David C. Glahn, Navil Sethna, Joseph Gonzalez-Heydrich, Jaymin Upadhyay

**Affiliations:** Boston Children’s Hospital; Brigham and Women’s Hospital; Boston Children’s Hospital; Boston Children’s Hospital; Ankara University; Boston Children’s Hospital; Mayo Family Pediatric Pain Rehabilitation Center; Mayo Family Pediatric Pain Rehabilitation Center; Brighton and Sussex Medical School; Brighton and Sussex Medical School; Boston Children’s Hospital; Boston Children’s Hospital; Boston Children’s Hospital; Boston Children’s Hospital; Boston Children’s Hospital; Boston Children’s Hospital; Boston Children’s Hospital; Boston Children’s Hospital; Boston Children’s Hospital

**Keywords:** Early-Onset Psychosis, Cognition, Prefrontal Cortex, Neuroimaging

## Abstract

Early-onset psychosis (EOP) is associated with marked cognitive impairment and poorer health-related and psychosocial outcomes compared to adult-onset illness, yet the neural mechanisms underlying these deficits during adolescence remain incompletely understood. Despite evidence implicating dorsolateral prefrontal cortex (dlPFC) dysfunction, the region’s functional and structural correlates in youth with EOP are not well characterized. Adolescents with EOP (N=31) and healthy controls (HC; N=20) completed NIH Toolbox cognitive assessments and PROMIS self-reports. Prefrontal function was assessed using functional near-infrared spectroscopy (fNIRS) during a Stroop task and at rest. Multimodal MRI, including resting-state fMRI, diffusion tensor imaging, and structural imaging, was used to examine connectivity and morphology of prefrontal and fronto-cerebellar circuits. Adolescents with EOP exhibited poorer cognitive performance across executive functioning domains compared to HC. fNIRS revealed reduced right dlPFC activation on a cognitive control paradigm (p=0.007) and increased resting-state connectivity between right dlPFC and ventrolateral PFC (p=0.02). Furthermore, resting-state fMRI showed increased dlPFC-striatal connectivity and reduced connectivity with cerebellar Crus I/II (p-FDR<0.05). White matter integrity of the superior longitudinal fasciculus correlated with dlPFC activation during task performance. Structural analyses identified reduced frontal cortical thickness and decreased cerebellar Crus II volumes (p-FWE<0.05) in patients with EOP, with frontal morphology associating with cognitive measures. In summary, cognitive impairment in adolescents with EOP is associated with convergent abnormalities in dlPFC function, fronto-striatal connectivity, and fronto-cerebellar structure. These findings support a model of disrupted prefrontal circuit maturation in EOP and highlight multimodal imaging markers with potential relevance for early identification and targeted intervention.

## Introduction

Early onset psychosis (EOP) is defined as the presence of psychotic symptoms before age 18 years. EOP, encompassing schizophrenia spectrum disorders and affective psychoses with psychotic features, has an estimated cumulative incidence of approximately 1% by age 18, though precise estimates vary by diagnostic criteria and population studied [6]. Although there is marked inherent heterogeneity in EOP, as diagnostic classifications in EOP are often provisional and evolve throughout illness trajectories [13], patients with EOP consistently exhibit broad cognitive impairments across domains including attention, working memory, verbal learning, and processing speed [23, 37]. EOP is associated with poorer premorbid function [41] and poor long-term prognosis [20], as well as more cognitive impairment (poorer sustained attention and executive functioning) [7, 31] compared to adult-onset psychosis. While aberrant neurobiological mechanisms underlying cognitive deficits in adult psychosis have been extensively characterized, the neurobiological underpinnings of cognitive dysfunction in EOP remain comparatively understudied, despite developmental vulnerability and evidence that this population experiences a more severe clinical course [8, 35, 38].

Neuroimaging studies have implicated disrupted cortical-subcortical-cerebellar circuitry in EOP, with aberrant functional connectivity in cortico-striato-thalamic and prefrontal-thalamic-cerebellar circuits correlating with both psychotic symptoms and cognitive impairments [17, 40, 45, 48]. Disruptions in subcortical and cerebellar regions are evident in the early stages of illness, but cortical–subcortical dysfunction may emerge later, suggesting alterations in prefrontal hubs that may reflect either progressive pathology or an exaggeration of typical developmental processes [32, 44]. Within this circuitry, the dorsolateral prefrontal cortex (dlPFC) has emerged as a central node in the pathophysiology of cognitive dysfunction in adults with psychosis [22, 39]. The dlPFC supports working memory and top-down cognitive control, with evidence suggesting hemispheric specializations where the left dlPFC is more strongly implicated in task setting and goal-oriented behavior while the right dlPFC supports monitoring and sustained attention [42]. Functional neuroimaging studies demonstrate reduced prefrontal cortex, specifically dlPFC activation, during cognitive tasks in adults with bipolar disorder and resting-state dlPFC connectivity disruptions have been observed, but similar findings in pediatric or adolescent psychosis patients are sparse [30]. Importantly, adolescence represents a critical neurobiological period for prefrontal maturation, with peak maturation occurring at age 14–16 years old, and high rates of synaptic pruning in the frontal cortex and dynamic shifts in excitatory-inhibitory metabolite balance supporting the development of higher-order cognition [1, 21, 26]. Cerebello-thalamocortical maturational processes, their impact on cognitive functioning and related psychopathological mechanisms of psychosis are increasingly recognized (Cao et al., 2018; Radhakrishnan et al., 2023), Disruptions in this circuitry at earlier age may have particularly profound consequences for functionality of the prefrontal cortex and lead to poorer cognition-related outcomes in individuals with EOP compared to cases of adult-onset psychosis [11, 18, 27].

The current study employs a novel neuroimaging technique functional near-infrared spectroscopy (fNIRS), combined with multimodal magnetic resonance imaging (MRI) including structural MRI, diffusion tensor imaging (DTI), and resting-state fMRI, to examine prefrontal dysfunction underlying cognitive impairments in adolescents with EOP. Compared with MRI, fNIRS offers several advantages, including its noninvasive, portable, and cost-effective nature [29, 34]. Importantly, fNIRS does not require participants to lie within an enclosed scanner and therefore minimizes the risk of claustrophobia or procedural distress, which is particularly relevant for adolescents with complex mood and psychological symptoms. fNIRS has been increasingly used to characterize prefrontal dysfunction in psychiatric disorders, with studies demonstrating reduced prefrontal activation during cognitive tasks in both schizophrenia and affective disorders [12, 47], but no prior studies have integrated fNIRS with multimodal MRI to characterize dlPFC-centered circuitry in adolescents with EOP.

This study aims to further elucidate functional and structural properties of the prefrontal cortex that relate to cognitive impairments in adolescents with EOP. fNIRS will assess prefrontal activation during cognitive tasks and MRI provides complementary assessment of fronto-cerebellar structural and functional connectivity, specifically probing fronto-cerebellar circuitry dysfunction. We hypothesize that adolescents with EOP will demonstrate reduced dlPFC prefrontal activation on fNIRS during cognitive task performance, along with fronto-cerebellar structural and functional circuitry disruptions, supporting a central role of dlPFC dysfunction in the neurobiological basis of cognitive deficits in EOP.

## Methods

### Study Participants

Boston Children’s Hospital (BCH) Institutional Review Board approval (IRB-P00045510) and written informed consent from the child and legal guardian was obtained. Male or female children with EOP and age-sex matched HC between the ages of 12 and 18 years were enrolled. Patients with EOP were primarily recruited through the BCH Outpatient Psychiatry Services, and HC were recruited from the BCH Research Participant Registry. Exclusion criteria for HC included acute illness or injury, history of psychiatric disorders, current DSM-5 diagnosis or any neurological illness. HC were also excluded from the study if they had a first-degree relative with a psychotic disorder.

#### Structured Interviews and Child-Reports

Study participants were evaluated with the Structured Clinical Interview for DSM-5 Axis I diagnosis Research Version (SCID-5-RV) to confirm EOP diagnosis or to identify exclusionary criteria in the HC cohort. Modules from the Kiddie Schedule for Affective Disorders and Schizophrenia (KSADS-PL DSM-5) and the Brief Psychiatric Rating Scale (BPRS), were also implemented while relevant clinical information including medication and psychiatry notes were reviewed. Participants also completed the Patient-Reported Outcomes Measurement Information System (PROMIS^®^) questionnaires reporting on cognitive functioning, psychological stress, anxiety and depressive symptoms [4]. Additionally, time of clinical diagnosis and medication history was documented through review of the individual’s medical records, and duration since the initial diagnosis of psychotic disorder and first antipsychotic medications were calculated.

#### Behavioral Assessment of Cognitive Functioning

Neurocognitive tests were performed on a NIH Toolbox platform [14], which included the Flanker Inhibitory Control and Attention Test (Executive Functioning/ Attention), Dimensional Change Card Sort Test (DCCS; Executive Functioning/ Shifting) and List Sorting Working Memory Test (Working Memory). Subject-specific age-corrected T-scores were calculated within NIH Toolbox, providing a relative score compared to the U.S. population for each neurocognitive test within the NIH Toolbox, which were subsequently also corrected for sex during statistical testing. Moreover, the color-word Stroop task paradigm was administered, as previously described [43]. Briefly, participants were visually presented with words and asked to identify correct probes for congruent or incongruent events. Percentage of error/correct trials and reaction times were logged.

#### Neuroimaging Acquisition

##### Functional Near-Infrared Spectroscopy

fNIRS data were collected in a quiet testing environment using a multichannel continuous-wave system (NIRx Medical Technologies, LLC) with wavelengths of 690 nm and 830 nm. A 28-channel cap, incorporating light sources along with standard- and short-separation detectors to reduce contributions from non-cerebral tissues (e.g., scalp and skull), was positioned over the prefrontal cortex to assess changes in oxygenated and deoxygenated hemoglobin. Channel placement followed the international 10–20 system to ensure consistent prefrontal coverage and channels corresponding to the PFC regions were identified based on the optode placement and anatomical channel registration provided by NIRx. fNIRS data were collected during an 8-minute resting-state scan during which participants were asked to fixate their gaze on a cross, and during the color-word Stroop paradigm as described above.

##### Whole-Brain, Structural and Functional MRI

MRI data were collected on a Siemens 3T Scanner (Siemens, Erlangen, Germany) with a 64-channel head coil at BCH. MRI acquisitions included structural T1-weighted imaging, diffusion tensor imaging (DTI), and resting-state functional MRI, with neuroimaging protocols adapted from The Human Connectome Project[16]. High-resolution anatomical MRI data were collected using a multi-echo magnetization-prepared rapid acquisition with gradient echo (MPRAGE) using the following parameters: 1 mm3, TR/TE1/TE2/TE3/TE4 = 2.53s/3.3ms/6.93ms/8.79ms/10.65ms, GRAPPA = 2. DTI data were collected using the following parameters: 1.5 mm isotropic, TR/TE = 3230ms/89.20ms, GRAPPA = 2, MB = 4, b = 0–3000s/mm2, 99 directions, 92 slices, and seven unweighted volume images (b = 0s/mm2). Resting-state fMRI data were collected using a gradient echo T2*-weighted echo-planar multi-band pulse sequence with 2 mm^3^ resolution, TR/TE = 800ms/37ms, 72 slices, GRAPPA = 2, multiband factor = 6. During resting-state fMRI acquisition, participants were asked to fixate on a cross for 6 minutes visually.

#### Neuroimaging Analysis

##### Functional Near-Infrared Spectroscopy

Analysis of fNIRS data was performed using Satori fNIRS (NIRX Software, Brain Innovation, BV, Netherlands). Before preprocessing, the task-based fNIRS data were trimmed by 10 seconds before the first and after the last trigger. The raw data was converted to optical density signals, and a signal quality index (SCI) threshold of 0.2 was set for channel rejection. Motion artifacts, including head movements, were corrected using spike removal methods, and temporal derivative distribution repair (TDDR) was applied to mitigate baseline shifts and residual spike artifacts. The signals were low and high bandpass-filtered at 0.01–0.2 Hz. Short-separation channels were incorporated during preprocessing to regress superficial physiological signals. After preprocessing, changes in light intensity were converted into oxyhemoglobin (Oxy-Hb) and deoxyhemoglobin (Deoxy-Hb) concentration changes using the Modified Beer–Lambert Law. General linear models were conducted to investigate the contrasting incongruent vs congruent task conditions HbO differences between healthy controls (HC) and patients with EOP, corrected for age and sex. Resting-state channel-to-channel Pearson cross-correlation matrices of Oxy-Hb were computed.

##### Resting-state fMRI

The MATLAB-based CONN-fMRI toolbox (version 18.b) in conjunction with SPM12 was used to analyze resting-state fMRI data. Data analysis included standard preprocessing and QA/QC, including motion correction, correction for susceptibility-induced distortions, denoising, realignment, slice-time correction, segmentation, spatial registration, and spatial smoothing with a 5-mm full-width-half-maximum (FWHM) kernel and band-pass temporal filtered (0.008, 0.09 Hz) [2]. One individual with EOP was excluded from further analysis due to excessive motion based on QA/QC outlier identification. Seed-based analyses were performed for left and right dlPFC ROIs separately, and a cluster-size threshold p < 0.05 p-FDR corrected for multiple comparisons and adjusted for age and sex results was applied. Spherical 8mm ROIs were created around center MNI coordinates 44,36,20 (right dlPFC) and − 44, 36,20 (left dlPFC) based on prior dlPFC peak coordinates[36].

##### Morphological Analysis

Automated cortical reconstruction and volumetric segmentation were performed using the FreeSurfer software package (version 7.1.1). T1-weighted MRI scans were motion-corrected and transformed to Talairach space. Non-brain tissue was removed through skull stripping, followed by intensity normalization to reduce bias field inhomogeneities and enable accurate cortical surface reconstruction. Cortical regions were parcellated according to the Desikan–Killiany atlas, which defines anatomical regions of interest (ROIs) based on gyral and sulcal landmarks. This processing pipeline generated measurements of cortical thickness (mm) for each ROI for both cortical and subcortical structures. Voxel-based morphometry analyses were conducted using a general linear model (GLM) framework to examine group differences, while controlling for age and sex. Correction for multiple comparisons was performed using Monte Carlo cluster-wise simulation, and statistical significance was determined using a cluster-corrected threshold of p < 0.05.

The Spatially Unbiased Infratentorial Template (SUIT) toolbox within Statistical Parametric Mapping (SPM12) was employed to conduct optimized voxel-based morphometry (VBM) analysis of the cerebellum [9, 10]. The structural images were aligned to the anterior commissure, followed by the segmentation of the cerebellum and brainstem. The cerebellum was then normalized to a cerebellar (SUIT-space) template, which has been shown to enhance alignment accuracy compared to standard whole-brain normalization methods [10]. All datasets were visually inspected to ensure accurate isolation and segmentation. Jacobian modulation was performed to prepare for VBM analysis, which included a two-sided t-test with covariates of sex, age, and eTIV, as well as correction for multiple comparisons with a family-wise error rate of p = 0.05. Volumes are reported as a percentage of the standardized cerebellar volume, which is based on an independent reference population.

##### Diffusion Weighted Imaging Analysis

Single-subject and group-level DTI were carried out using FMRIB Software Library (www.fmrib.ox.ac.uk/fsl). Each subject’s dataset was corrected for eddy-current distortion and head motion using an automated affine registration algorithm with the skull-stripped structural image as the reference volume. A least squares fit of the tensor model was used to calculate a diffusion tensor for each voxel and the eigenvalues of each tensor, representing the diffusion directions, and fractional anisotropy (FA), mean diffusivity (MD), axial diffusivity (AD) and radial diffusivity (RD) values were calculated. Subsequently, tract-based spatial statistics were performed to determine group-level differences in FA between patients with EOP and HC, and multiple comparison correction was applied by voxel-wise permutation (5000 permutations) testing, and clusters were deemed significant at a threshold of p < 0.05. The superior longitudinal fasciculus (SLF) subdivisions I, II and III defined by the XTRACT HCP Probabilistic Tract Atlas were investigated as they connect the frontal lobe to other cortical regions. Each study participant was co-registered to a template brain and tractography measures were extracted for SLF subdivisions.

##### Statistical Analysis

Non-parametric linear models with covariate adjustments for age and sex were used to assess group differences between EOP and HC groups. For imaging measures that were modeled and corrected for age, sex and intracranial volume as well as multiple comparisons within the analysis pipeline, Cohen’s d was calculated to quantify effect sizes for each pairwise group comparison. Nonparametric partial correlation (rank-based) controlling for age and sex were computed across HC and EOP cohorts, with adjustment for chlorpromazine equivalent dose (CPZeq) for associations within the EOP group. All imaging-derived measures underwent rigorous multiple comparisons correction within their respective processing pipelines, correlation analyses were restricted to a small number of a priori defined cognitive task outcomes, so additional correction for multiple testing was not applied to correlation analyses to avoid undue inflation of Type II error.

## Results

### Participant Characteristics

EOP patient (N = 31) and HC (N = 20) demographics are shown in Table 1, and additional cohort sizes and demographics included per assessment are stated in **Supplemental Table 1.** Psychotic diagnoses included schizophrenia (32%), schizoaffective disorder (19%), bipolar I disorder with psychotic features (13%), depressive disorder with psychotic features (13%) and other specified psychotic disorders (i.e., delusional disorder, brief psychotic disorder) (22%). Out of the 31 EOP patients, 30 were on antipsychotic treatment, with CPZeq ranging at time of enrollment from 50 to 1375 mg (mean 344.4 ± SD 300.8 mg). Additionally, 17 individuals were on anti-depressants and 8 were on mood stabilizers (not including atypical mood-stabilizing antipsychotics). Age at initial diagnosis of psychotic disorder ranged from 5 to 17 years old (mean 9.9 ± SD 3.6), with duration since initial diagnosis ranging from 6 months to 14 years (mean 6.1 ± SD 3.8). Age at first antipsychotic medication ranged from 4 to 18 (mean 11.1 ± SD 3.9), with duration ranging from 6 months to 12 years (mean 5.0 ± SD 3.8). Five patients with EOP had contraindications to MRI.

### Self-reports of Cognitive and Psychological Symptoms

**Figure 1:**
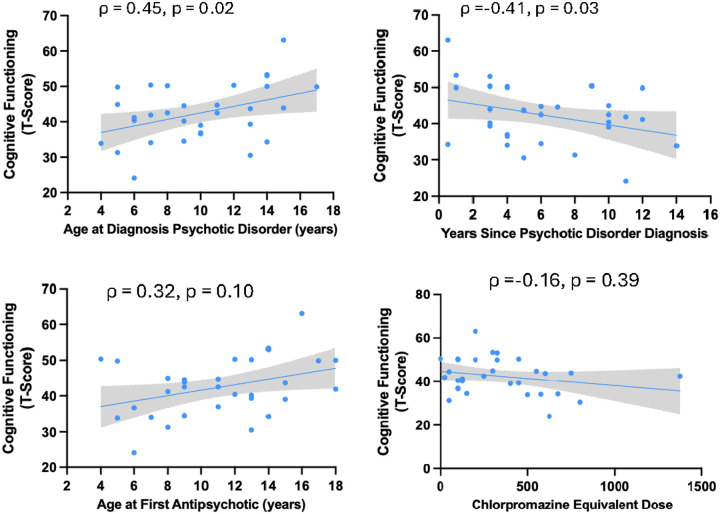
Lower self-reported cognitive functioning T-scores on the PROMIS questionnaire associated with a younger age at diagnosis in adolescents with EOP and duration since initial diagnosis, but not with age at first antipsychotic medication and CPZeq dose at time of enrollment. Corrected for sex, age and CPZeq; Correlation between cognitive functioning and CPZeq score corrected only for age and sex.

**Figure 2:**
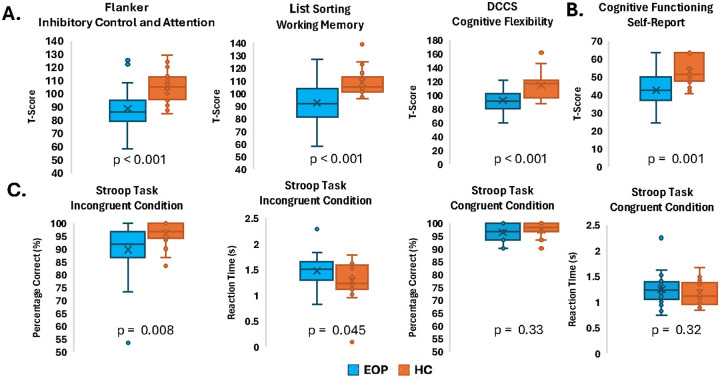
(A)**Patient with EOP show** poorer cognitive performance on the NIH Toolbox Flanker inhibitory control and attention, list sorting working memory and dimensional card change sort (DCCS) task, evidenced by lower T-Scores compared to HC (corrected for age and sex). (B) Patients with EOP reported worse cognitive functioning on the PROMIS cognitive functioning questionnaire (Short-Form v. 7a) compared to HC. (C) Patients with EOP perform worse (fewer correct responses) and demonstrate longer reaction times (seconds) on the incongruent condition of the color-word Stroop task are observed in patients with EOP compared to HC, but not on the congruent condition.

Individuals with EOP reported that they experience more cognitive difficulties, psychological stress, anxiety and depressive symptoms compared to HC (Table 1, Fig. 2B). Among EOP patients, poorer self-reported cognitive functioning was associated with a younger age at diagnosis and longer illness duration (Fig. 1). Conversely, no significant associations were observed between self-reported cognitive functioning and age at first antipsychotic use or CPZeq dose at time of enrollment.

### Cognitive Task Performance

Age-corrected T-scores on NIH toolbox Flanker, list sorting and dimensional card change sort tasks were significantly lower in adolescents with EOP compared to HC (Fig. 2A). Furthermore, the percentage of correct responses was significantly lower, and reaction times were higher for the incongruent condition in patients with EOP compared to HC, whereas no significant differences were observed for the congruent condition (Fig. 2C).

### fNIRS Task-based and Resting-State Beta Oxygenation

**Figure 3:**
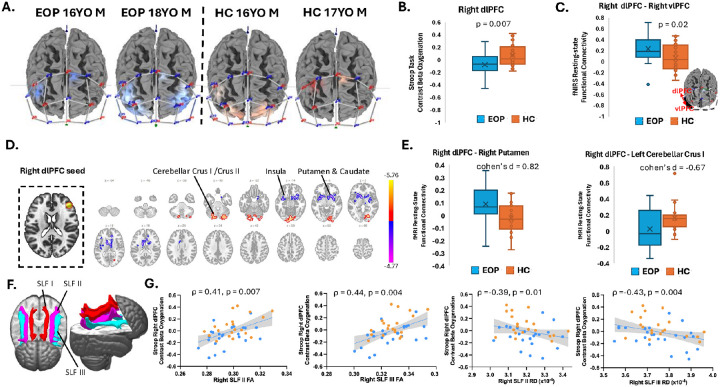
(A) fNIRS-based contrast (incongruent vs. congruent) activation maps during the Stroop task. Blue represents lower activation, and red represents higher activation, with (B) EOP participants showing decreased prefrontal activation compared to HC in the right dorsolateral prefrontal cortex (dlPFC), corrected for age and sex. (C) Higher fNIRS resting-state functional connectivity between channels in the right dlPFC and right vlPFC were observed in patients with EOP compared to HC. (D) fMRI resting-state seed-based connectivity from a right dlPFC seed ROI (8mm spherical ROI with center MNI coordinates 44, 36, 20 (Right) and −44, 36, 20 (Left) demonstrates clusters of reduced connectivity to bilateral cerebellar Crus I and Crus II and increased connectivity to the bilateral insula, striatum and the right occipital pole in patients with EOP compared to HC (cluster wise p-FDR < 0.05, corrected for age and sex). (E) Parameter estimates for right dlPFC to right Putamen and left cerebellar crus I are depicted. (F) SLF I, II and II subdivisions, as defined by the XTRACT HCP Probabilistic Tract Atlas, visualized on a template brain. (G) Correlations of stroop task contrast beta oxygenation values correlated with FA values of the right SLF II (all: ρ = 0.41, p = 0.007, EOP: ρ = 0.36, p = 0.11, HC: ρ = 0.36, p = 0.14) and SLF III (all: ρ = 0.44, p = 0.004, EOP: ρ = 0.47, p = 0.03, HC: ρ = 0.34, p = 0.17), as well as RD values of the right SLF II (all: ρ = −0.39, p = 0.01, EOP: ρ = −0.22, p = 0.34, HC: ρ = −0.44, p = 0.06) and right SLF III (all: ρ = −0.43, p = 0.004, EOP: ρ = −0.36, p = 0.11, HC: ρ = −0.50, p = 0.03) are shown.

On the color-word Stroop task, lower fNIRS-based beta oxygenation values for the incongruent vs. congruent condition contrast were observed in the right dlPFC in patients with EOP compared to HC (Fig. 3A–B), while other prefrontal regions did not show significant group differences. Oxygenation values in the dlPFC were positively correlated to self-reported cognitive functioning on PROMIS across participants (ρ = 0.35, p = 0.02) but not within EOP patients (ρ = 0.02, p = 0.9) or HC (ρ = 0.05, p = 0.84) cohorts.

fNIRS-based prefrontal resting-state analysis revealed increased connectivity between the right dlPFC and right vlPFC in individuals with EOP compared to HC (Fig. 3C). Resting-state connectivity values between other channels did not differ significantly.

### Resting-State Functional Connectivity of the dlPFC

Seed-based connectivity with the dlPFC regions, guided by fNIRS findings, demonstrated higher resting-state fMRI connectivity from the right dlPFC to bilateral striatum (putamen, caudate, nucleus accumbens and thalamus) and insula, while lower connectivity to the bilateral cerebellar Crus I and right occipital pole was observed in patients with EOP compared to HC (Fig. 3D–E). Furthermore, EOP patients showed increased connectivity from the left dlPFC to the pre- and postcentral gyrus compared to HC (**Supplemental Fig. 1**).

### White-Matter Structural Connectivity of the Prefrontal Cortex

Voxel-level comparisons revealed no significant group differences in FA, MD, AD or RD values of the SLF subdivisions (Fig. 3F) between EOP and HC. Significant correlations were observed between FA values and RD values of the right SLF II and III and right dlPFC oxygenation on the contrasted conditions of the Stroop task across study participant (Fig. 3G). Left SLF II RD values and SLF III FA and RD values also correlated with right dlPFC oxygenation (**Supplemental Table 2)**, but not bilateral SLF I FA or RD nor left SLF II FA values. No significant associations were observed for AD or MD values of SLF subdivisions and right dlPFC beta oxygenation values on Stroop task.

### Prefrontal Cortex Morphology

**Figure 4:**
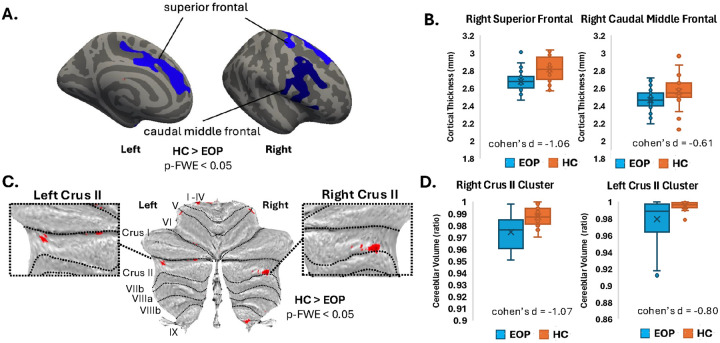
(A) Frontal regions based on the Desikan-Killiany atlas showing lower cortical thickness (mm) in patients with EOP compared to HC are overlayed on an MNI freesurfer original surface template. These include the superior frontal gyrus (left p = 0.02, p_fdr_ = 0.18; right p = 0.01, p_fdr_ = 0.09), middle frontal gyrus (left p = 0.05, p_fdr_ = 0.24), inferior triangularis (right p = 0.04, p_fdr_ = 0.15), inferior operculum (right p = 0.04, p_fdr_ = 0.16), orbital H-shaped (not depicted; right p = 0.01, p_fdr_ = 0.09) and precuneus (left p = 0.02, p_fdr_ = 0.18). (B) Decreased cortical thickness in the right middle frontal and right superior frontal regions in patients with EOP compared to HC is shown. (C) Clusters of decreased cerebellar volume identified by voxel-based morphological analysis in EOP patients compared to HC are located in bilateral Crus II, left VI and right IX, overlaid on the SUIT cerebellar flatmap (FWE-corrected, *p* < 0.05, cluster-corrected). (**D)** Box plots display cerebellar volumetric reductions in the left and right Crus II among patients with EOP compared to HC.

Voxel-based morphological analysis revealed reduced cortical thickness in the bilateral superior frontal gyri and the right caudal middle frontal gyrus (Fig. 4A–B), as well as in bilateral mid temporal gyri, left supramarginal gyrus and right inferior parietal gyrus (cluster-wise p-FWE < 0.05) in patients with EOP compared to HC. Right superior frontal and caudal middle frontal thicknesses associated significantly with self-reported cognitive functioning on PROMIS, Flanker Task performance and fNIRS Stroop task oxygenation in the dlPFC (channel 17) across all study participants, but not within EOP or HC cohorts separately (**Supplemental Table 3).**

### Cerebellar Morphology

Cerebellar morphometry analyses identified clusters of significantly reduced cerebellar volume in patients with EOP compared to HC after correcting for age, sex and eTIV (Fig. 4C). Bilateral volume reductions were observed in bilateral Crus II (Fig. 4D), Left VI and right V and IX in the EOP cohort compared to HC. Cerebellar volume of these clusters did not significantly associate with cognitive task performance in EOP patients.

## Discussion

This study used a multimodal neuroimaging approach to investigate neural underpinnings of cognitive impairment in adolescents with EOP. Compared to healthy controls, adolescents with EOP demonstrated poorer cognitive performance across multiple domains, including inhibitory control and attention, working memory and executive functioning. These deficits were accompanied by decreased right dlPFC task-based oxygenation and increased resting-state functional connectivity measured with fNIRS. Altered dlPFC connectivity with the striatum and cerebellar Crus II were revealed using resting-state fMRI and structural integrity of the right SLF was positively associated with dlPFC activity during Stroop-task performance. Furthermore, morphological analyses showed reduced frontal cortical thickness and decreased cerebellar Crus II volume in individuals with EOP. Overall, these findings suggest that disruptions in prefrontal and fronto-cerebellar circuitry contribute to cognitive deficits in EOP. These results support a model in which disruptions of dlPFC-centered circuits, particularly fronto-striatal and fronto-cerebellar pathways, play a role in cognitive dysfunction in EOP.

The current findings of dlPFC dysfunction related to cognitive deficits in adolescents with EOP align with previous research in both pediatric and adult patients. Neuroimaging studies of adolescents with psychosis spectrum symptoms and with clinical high risk for psychosis demonstrated reduced dlPFC activation during working memory and conflict-related tasks compared to healthy controls [5, 46]. These findings may point to impaired capacity to recruit dlPFC resources in response to increasing cognitive demand. Additionally, structural imaging has revealed gray matter volume reductions in the dlPFC among adolescents with psychosis spectrum symptoms [33]. In adults with psychosis, dlPFC dysfunction has been well characterized and recognized as a potential therapeutic target [39]. Collectively, these findings indicate that dlPFC dysfunction is a shared neurobiological substrate across the psychosis spectrum, present from early developmental stages through adulthood, and closely linked to cognitive deficits. The convergence of functional hypoactivation, hyperconnectivity, and structural alterations may reflect disrupted developmental tuning of prefrontal networks during adolescence.

While the current cohort demonstrated an association between self-reported cognitive functioning and both age of onset and psychotic symptom duration but not with age of antipsychotic or CPZeq at time of enrollment. Prior longitudinal research in adolescents and early adults has shown that dlPFC deficits are present at psychosis onset regardless of age, but the developmental trajectory paralleled healthy controls, suggesting development continues rather than arrests [25]. Together, these findings suggest that cognitive functioning deficits in psychosis may reflect core features of the underlying pathophysiology, rather than being attributable solely to illness chronicity or exposure to antipsychotic medication.

Given the observed functional connectivity abnormalities between the dlPFC and cerebellar Crus I we extended our analyses to include cerebellar morphology, in order to better characterize potential structural contributions of this fronto-cerebellar network to cognitive impairments in EOP. Volumetric reductions in cerebellar Crus II were observed in the current cohort of adolescents with EOP. Previous investigations of cerebellar volume in EOP and in individuals at clinical high risk for psychosis have similarly reported abnormalities in Crus II, while also identifying volumetric reductions in Crus I [44]. The consistent involvement of Crus II may reflect its strong integration within frontoparietal and default mode networks, which are consistently implicated in psychosis [3, 15, 28]. In contrast, the absence of Crus I alterations may be due to factors such as a CHR cohort, percentage of males and females, or different cohort ages (12–18 vs. 5–17 years old), although limited evidence exists regarding developmental trajectories and sex differences between Crus I and II [19, 24].

Several study limitations should be noted. The modest sample size of the current study, further reduced by MRI contraindications in five participants, limits the generalizability of our findings. The cross-sectional design cannot determine whether prefrontal abnormalities precede or follow illness onset. The inherent diagnostic heterogeneity of EOP(schizophrenia spectrum vs. affective psychoses) may obscure diagnosis-specific patterns and the current cohort size does not allow for further differentiation of diagnostic groups. Additionally, high rates of psychiatric comorbidity in EOP complicate attribution of cognitive phenotypes specifically to psychosis. While our statistical analysis adjusted for antipsychotic medication dosage, the majority of patients were on antipsychotics, and other medications or prior treatment exposures may yet negatively influence cognitive, behavioral or neuroimaging measures. However, for this severely affected population, antipsychotic treatment is clinically necessary despite its potential confounding effects on cognitive and neuroimaging measures, as withholding medication would likely result in worse clinical outcomes. Lastly, while fNIRS and fMRI offer complimentary insights into prefrontal functionality, fNIRS cannot capture subcortical connectivity, thus limiting direct comparison across modalities. Future studies incorporating longitudinal designs and complementary neurophysiological methods in a larger sample of EOP patients to further elucidate the neurobiological underpinnings of cognitive impairments in adolescents with EOP.

The integration of emerging neuroimaging techniques, such as fNIRS, offers promising opportunities for improving early detection and informing interventions targeting cognitive dysfunction, thereby potentially mitigating their long-term impact. The observed reductions in dlPFC oxygenation during the Stroop task in adolescents with EOP were detectable with the use of high temporal resolution of fNIRS, which allows measurement of rapid, task-evoked cortical hemodynamic responses. In contrast, the comparatively lower temporal resolution on fMRI might have obscured such condition-specific fluctuations. Furthermore, fNIRS acquisition was feasible even for individuals that were contraindicated for MRI (e.g., due to claustrophobia or metal implants) thereby broadening inclusion and enhancing cohort representativeness. fNIRS could serve as a scalable, clinically feasible tool for assessing prefrontal dysfunction in youth populations.

The current findings strengthen the growing body of evidence linking prefrontal abnormalities with cognitive impairments in adolescents with EOP. Using a multimodal neuroimaging approach, this study provides a foundation for future large-scale, multi-site investigations to identify both mechanistic and clinical predictors of cognitive symptom severity. A deeper understanding of these neurobiological processes will be crucial for improving clinical recognition of cognitive impairments and guiding the development of targeted interventions tailored to distinct neuropsychiatric symptoms profiles in EOP patients. Longitudinal larger-scale future studies that integrate advanced neuroimaging and cognitive assessments hold potential for advancing tailored approaches to improve cognitive outcomes in adolescents with EOP.

## Supplementary Material

Supplementary Files

This is a list of supplementary files associated with this preprint. Click to download.

- SupplementalTablesandFigCaptions.docx

## Figures and Tables

**Table 1:** 

Age	EOP	HC	p-value^2^
	N = 31^1^	N = 20^1^	
	17.0 (2.6)	15.9 (2.7)	0.10
**Sex**			
Male	19 (61%)	12 (60%)	
Female	12 (39%)	8 (40%)	
**PROMIS, Cognitive Function**	42.5 (13.1)	51.4 (15.1)	< 0.001
**PROMIS, Psych Stress Experiences**	60.9 (14.5)	51.2 (11.9)	0.006
**PROMIS, Anxiety**	55.7 (17.7)	45.4 (9.9)	< 0.001
**PROMIS, Depressive Symptoms**	57.5 (16.2)	48.1 (8.6)	0.002
**BPRS, Total**	35.0 (10.5)	18.0 (0.0)	< 0.001
**BPRS, Positive**	6.0 (3.0)	4.0 (0.0)	< 0.001
**BPRS, Negative**	4.0 (6.0)	3.0 (0.0)	< 0.001
**BPRS, Affectivity**	7.0 (5.0)	4.0 (0.0)	< 0.001
**BPRS, Resistance**	7.0 (4.0)	3.0 (0.0)	< 0.001
**BPRS, Activation**	4.0 (3.0)	3.0 (0.0)	< 0.001

1 Median (IQR); n (%)

2 Kruskal-Wallis rank sum test

## Data Availability

Data will be made available upon reasonable request.
